# Association between Glu504Lys Polymorphism of ALDH2 Gene and Cancer Risk: A Meta-Analysis

**DOI:** 10.1371/journal.pone.0117173

**Published:** 2015-02-13

**Authors:** Qiang Cai, Jian Wu, Qu Cai, Er-Zhen Chen, Zhao-Yan Jiang

**Affiliations:** 1 Department of Surgery, Shanghai Institute of Digestive Surgery, Ruijin Hospital, Shanghai JiaoTong University School of Medicine, Shanghai, China; 2 Department of Emergency, Ruijin Hospital, Shanghai JiaoTong University School of Medicine, Shanghai, China; Duke Cancer Institute, UNITED STATES

## Abstract

**Background:**

The association of the aldehyde dehydrogenases-2 (ALDH2) Glu504Lys polymorphism (also named Glu487Lys, or rs671) and cancers has been investigated. This meta-analysis aims to comprehensively assess the influence of this polymorphism on the overall cancer risk.

**Methods:**

Eligible publications were retrieved according to inclusion/exclusion criteria and the data were analyzed using the Review Manager software (V5.2).

**Results:**

A meta-analysis based on 51 case-control studies consisting of 16774 cases and 32060 controls was performed to evaluate the association between the ALDH2 Glu504Lys polymorphism and cancer risk. The comparison of genotypes Lys+ (Lys/Lys and Lys/Glu) with Glu/Glu yielded a significant 20% increased cancer risk (OR = 1.20, 95%CI: 1.03–1.39, *P* = 0.02, *I^2^* = 92%). Subgroup analysis by cancer type indicated a significantly increased UADT cancer risk (OR = 1.39, 95%CI: 1.11–1.73, *P* = 0.004, *I^2^* = 94%) in individuals with the Lys+ genotypes. Subgroup analysis by country indicated that individuals from Japan with the Lys+ genotypes had a significant 38% increased cancer risk (OR = 1.38, 95%CI: 1.12–1.71, *P* = 0.003, *I^2^* = 93%).

**Conclusions:**

Our results indicated that the ALDH2 Glu504Lys polymorphism is a susceptible loci associated with overall cancers, especially esophageal cancer and among Japanese population.

## Introduction

Based on available epidemiological data, alcohol ingestion is shown to be carcinogenic to humans and causally related with liver, colorectal, female breast and upper aerodigestive tract (UADT) cancers [1]. Approximately 3.6% of all cancer-related cases and 3.5% of all cancer-related deaths worldwide are related to chronic alcohol drinking [2]. Alcohol in humans is oxidized to acetaldehyde, which interferes DNA synthesis and repair and consequently results in tumor development [3].

Aldehyde dehydrogenase-2 (ALDH2) is expressed in the liver as well as gastrointestinal tract. It belongs to a low-Km mitochondrial ALDH and is the second enzyme to eliminate most of the acetaldehyde generated during alcohol metabolism in vivo [4]. Human ALDH2 gene is located on chromosome 12q24 and the polymorphisms of ALDH2 gene would affect the blood acetaldehyde concentrations after alcohol consumption [5]. The Glu504Lys polymorphism (also named Glu487Lys, or rs671 has been the most commonly studied [6]. The exact position of the variant is 457 of NP_001191818.1 and 504 of NP_000681.2. The glutamate of this polymorphism is corresponding to *1 allele, and lysine corresponding to *2 allele. Such a polymorphism (Glu to Lys, or G to A, or *1 to *2) was reported to have decreased activity of ALDH2 enzyme and cause much higher blood levels of acetaldehyde, which is highly prevalent among East Asians [7].

Therefore, it is hypothesized that the genetic polymorphism in ALDH2 gene may be strongly correlated with the susceptibility to cancer, and a number of studies have investigated the association between ALDH2 Glu504Lys polymorphism and cancer risk. Most of the studies focused on esophageal cancer, followed by colorectal cancer, head and neck cancer, etc. In a meta-analysis by Yang et al [8], ALDH2 504Lys allele was found to increase the risk of esophageal cancer at all levels of exposure to ethanol and acetaldehyde after drinking. On contrary, another meta-analysis by Zhao et al [9] showed reduced risk for colorectal cancer associated with ALDH2 504lys allele carriers. Considering the contradictory results of the previous studies on ALDH2 Glu504Lys polymorphism with different cancers, we conducted the present meta-analysis to evaluate the relation of ALDH2 Glu504Lys polymorphism with the overall cancer risk.

## Materials and Methods

### Search strategy

Publications were searched via the PubMed bibliographical database with the last update as of 30 April, 2014. The following keywords and MeSH terms were used: [“aldehyde dehydrogenase 2” or “ALDH2”] and [“polymorphism” or “genetic polymorphism” or “mutation” or “variation” or ”variant” or “single nucleotide polymorphism” or “SNP”] and [“cancer” or “malignant tumor” or “malignant neoplasm”]. As a prerequisite, only studies published in English were identified. All eligible studies were retrieved and the full text of the articles was examined to make sure the data of interest were included. In addition, if multiple reports from the same patients were found, only the publication with the most complete data set was included. If more than one ethnic population or cancer type were included in one article, data were extracted separately for each ethnic population or cancer type whenever possible.

### Inclusion and exclusion criteria

Studies that we identified were required to meet the following criteria: (1) study on the evaluation of the ALDH2 Glu504Lys polymorphism and cancer risk; (2) case-control study that used either population- or hospital-based designs; (3) study that contained complete information about all genotype frequency. Studies were excluded if they were case-only studies, review articles, or reports without usable data.

### Extracted information

Two investigators (QC and JW) independently extracted the following information from all selected articles: first author, year of publication, country of origin, population ethnicity, study design (population- or hospital-based), cancer type, genotyping information (number of genotypes, genotype distribution in cases and controls). Ethnic backgrounds were categorized as Asian, African or Mixed (composed of different ethnic groups). Cancers of oral cavity, oropharynx, hypopharynx, larynx, esophagus and stomach were defined as upper aerodigestive tract (UADT) cancer [10].

### Statistical analysis

Before estimating the relationship between the ALDH2 Glu504Lys polymorphism and cancer risk, we tested whether the genotype frequencies of the controls were in Hardy-Weinberg equilibrium (HWE) using a *χ* ^2^ test (P>0.05) [11].

The strength of the association between the ALDH2 Glu504Lys polymorphism and cancer risk was measured by odds ratios (ORs) with their 95% confidence intervals (95%CI). The statistical significance of pooled ORs was assessed by the Z-test. Heterogeneity was assessed by the *I* ^2^ statistic, which was documented for the percentage of the observed between-study variability due to heterogeneity rather than chance with the ranges of 0 to 100% [*I* ^2^ = 0–25%, no heterogeneity; *I* ^2^ = 25–50%, moderate heterogeneity; *I* ^2^ = 50–75%, large heterogeneity; *I* ^2^ = 75–100%, extreme heterogeneity] [12]. When the *Q* test was significant (P<0.05) or *I* ^2^>50%, indicating the presence of heterogeneity, a random-effects model (the DerSimonian & Laird method) was used [13]; otherwise, the fixed-effects model (the Mantel-Haenszel method) was used [14]. Sensitivity analysis was performed by excluding the studies that the genotype distribution in controls was not in HWE or which did not provide the three genotypes in controls to evaluate the stability of the results. Statistical analysis was conducted using the software Review Manager (V5.2) for Mac Os X.

Publication bias was evaluated by using the fail-safe number (N_fs_) with the significance set at 0.05 for each meta-comparison. If the calculated N_fs_ value was smaller than the number of observed studies, the result might run the risk of having publication bias. We calculated the N_fs_0.05 according to the formula N_fs_0.05 = (ΣZ/1.64)^2^-k, where k is the number of included articles [15].

## Results

### Studies and population

**Figure 1:**
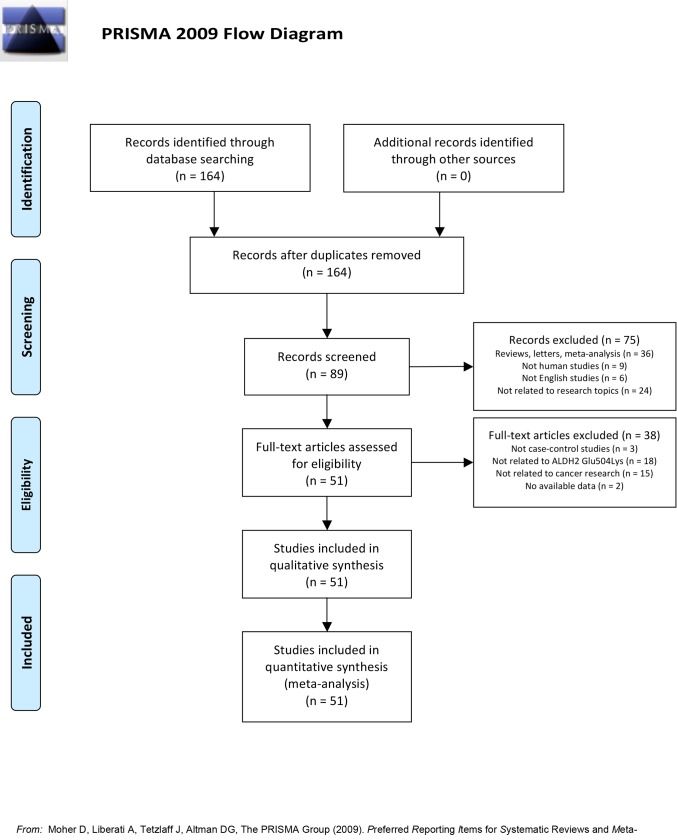
Flow diagram of search strategy and study selection.

Initially, we identified 164 related articles. The titles and abstracts of all articles were reviewed and 75 articles were excluded; full texts were also reviewed and 38 articles were further excluded. Finally, 51 case-control studies with a total of 16774 cases and 32060 controls were included in this meta-analysis. A diagram schematizing the selection process is presented in Fig. 1. Cancers were confirmed pathologically or histologically in most articles. Because the studies of Miyasaka et al [16] and Li et al [17] each included separate analysis of two cancer types and population, we treated them separately. As shown in Table 1, there are 53 case-control studies from 51 publications in the meta-analysis. The genotype distribution in the controls of all studies was in agreement with the HWE except for 11 studies, in 8 studies allele distributions were not in HWE [16–23] and in 3 studies the *P*_HWE_ values were not available [24–26]. The detailed characteristics of the studies are shown in the S1 Table.

**Table 1:** Characteristics of studies included in ALDH2 Glu504Lys polymorphism and cancer risk.

**First author**	**Country**	**Ethnicity**	**Cancer typers**	**Study design**	**Sample size**		**HWE**
					**Case**	**Control**
Matsuo 2013 [27]	Japan	Asian	UADT	HB	696	1372	Yes
Gao 2013 [28]	China	Asian	UADT	PB	2104	2265	Yes
Wu 2013 [29]	China	Asian	UADT	PB	801	1027	Yes
Chiang 2012 [18]	China	Asian	Colorectal	PB	103	545	No
Matsuo 2012 [30]	Japan	Asian	UADT	HB	251	759	Yes
Gu 2012 [31]	China	Asian	UADT	HB	380	378	Yes
Li 2011 [19]	China	Asian	UADT	HB	226	246	No
Wang 2011 [32]	China	Asian	UADT	PB	81	163	Yes
Shin 2011 [33]	Korea	Asian	UADT	HB	445	370	Yes
Ji 2011 [34]	Korea	Asian	UADT	HB	225	301	Yes
Miyasaka 2010 [16]	Japan	Asian	Pancreatic	PB	187	2070	Yes
Miyasaka 2010 [16]	Japan	Asian	Colon	PB	48	252	No
Tanaka 2010 [20]	Japan	Asian	UADT	HB	1071	2761	No
Oikawa 2010 [24]	Japan	Asian	UADT	HB	62	62	NA
Cao 2010 [35]	China	Asian	UADT	PB	382	382	Yes
Oze 2010 [36]	Japan	Asian	UADT	HB	585	1170	Yes
Sangrajrang 2010 [62]	Thailand	Asian	Breast	HB	561	486	Yes
Park 2010 [65]	Japan	Asian	Lung	HB	718	1416	Yes
Ding 2009 [21]	China	Asian	UADT	PB	191	221	No
Eom 2009 [25]	Korea	Asian	Lung	PB	387	387	NA
Cui 2009 [37]	Japan	Asian	UADT	Combined	1066	2762	Yes
Yang 2009 [52]	China	Asian	Colorectal	HB	426	785	Yes
Kawase 2009[63]	Japan	Asian	Breast	HB	456	910	Yes
Kanda 2009 [66]	Japan	Asian	Pancreatic	HB	160	1600	Yes
Li 2008 [17]	South Afica	Afican	UADT	PB	141	174	Yes
Li 2008 [17]	South Afica	Mixed	UADT	PB	96	94	No
Ding 2008 [22]	China	Asian	Hepatocelluar	PB	208	207	No
Guo 2008 [39]	China	Asian	UADT	PB	80	480	Yes
Gao 2008 [53]	China	Asian	Colorectal	PB	190	222	Yes
Yang 2007 [38]	China	Asian	UADT	PB	191	198	Yes
Hiraka 2007 [40]	Japan	Asian	UADT	HB	239	715	Yes
Asakage 2007 [44]	Japan	Asian	UADT	PB	96	642	Yes
Yin 2007 [54]	Japan	Asian	Colorectal	PB	685	778	Yes
Hashimoto 2006 [41]	Japan	Asian	UADT	Combined	192	192	Yes
Chen 2006 [42]	China	Asian	UADT	HB	330	592	Yes
Cai 2006 [43]	China	Asian	UADT	PB	205	394	Yes
Sakamoto 2006 [59]	Japan	Asian	Hepatocelluar	HB	209	275	Yes
Matsuo 2006 [56]	Japan	Asian	Colorectal	HB	257	768	Yes
Yang 2005 [45]	Japan	Asian	UADT	HB	165	494	Yes
Wu 2005 [46]	China	Asian	UADT	HB	134	237	Yes
Otani 2005 [55]	Japan	Asian	Colorectal	HB	106	224	Yes
Kuriki 2005[57]	Japan	Asian	Colorectal	HB	126	238	Yes
Munaka 2003 [26]	Japan	Asian	Hepatocelluar	HB	78	138	NA
Choi 2003 [64]	Korea	Asian	Breast	HB	307	360	Yes
Boonyaphiphat 2002 [23]	Thailand	Asian	UADT	HB	202	261	No
Yokoyama 2002 [47]	Japan	Asian	UADT	PB	234	634	Yes
Matsuo 2002 [58]	Japan	Asian	Colorectal	HB	141	231	Yes
Yu 2002 [60]	China	Asian	Hepatocelluar	PB	132	134	Yes
Matsuo 2001 [48]	Japan	Asian	UADT	HB	102	241	Yes
Chao 2000 [49]	China	Asian	UADT	HB	29	105	Yes
Takeshita 2000 [61]	Japan	Asian	UADT	HB	102	125	Yes
Katoh 1999 [50]	Japan	Asian	UADT	HB	92	147	Yes
Hori 1997 [51]	Japan	Asian	UADT	PB	93	70	Yes

UADT, upper aerodigestive tract; HB, hospital based; PB, population based; Combined, studies conducted on both population- and hospital-based control group; HWE, Hardy-Weinberg equilibrium; NA, not available.

### Pooled analysis

**Figure 2:**
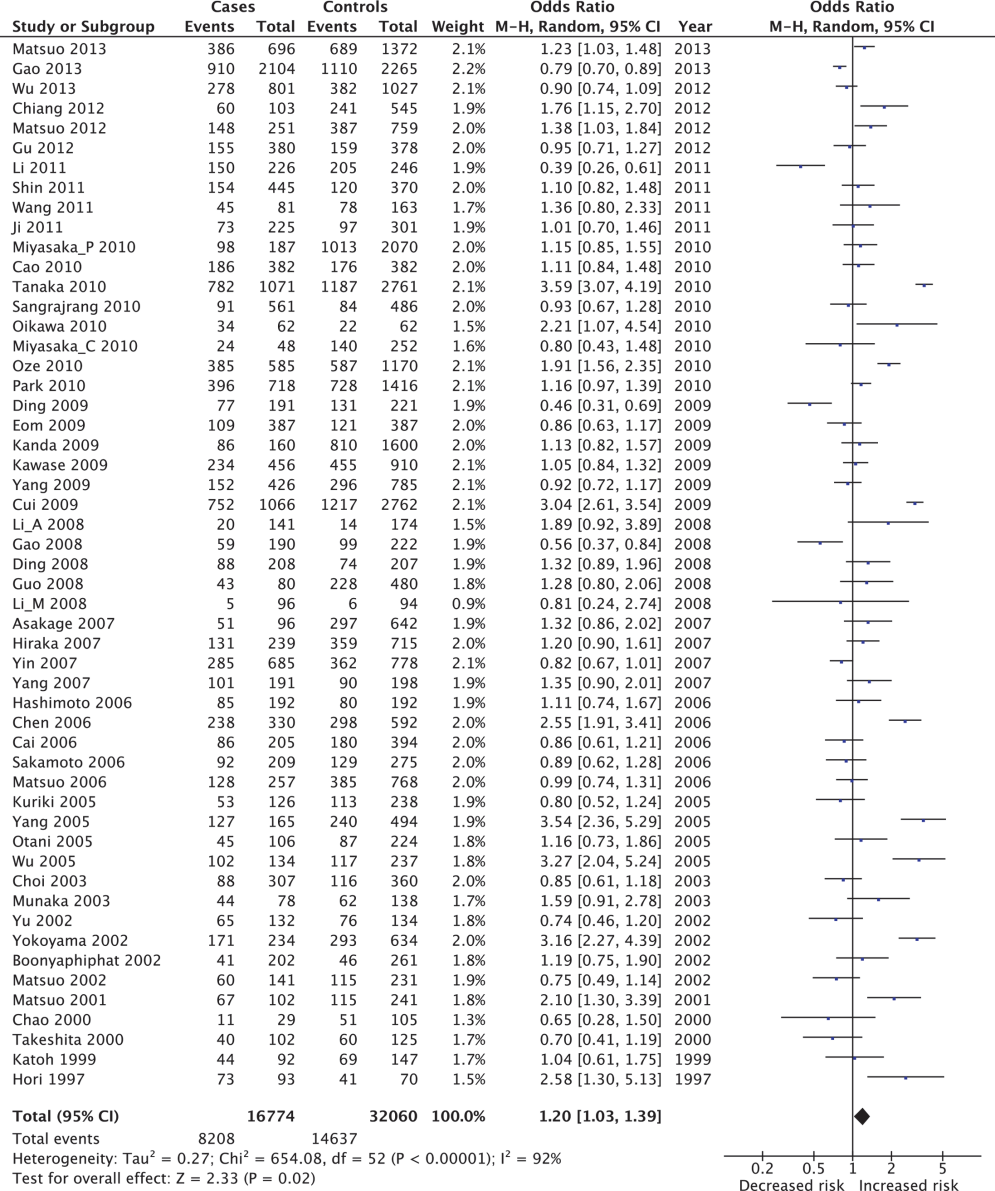
Forest plot showed the overall cancer risk in association with the polymorphism of ALDH2 gene Glu504Lys polymorphism. In the genotypic model, the comparison of Lys+ with Glu/Glu genotype generated a significant 20% increased cancer risk (OR = 1.20, 95%CI: 1.03–1.39, *P* = 0.02, *I^2^* = 92%).

In the genotypic model, the comparison of Lys+ with Glu/Glu genotype generated a significant 20% increased cancer risk (OR = 1.20, 95%CI: 1.03–1.39, *P* = 0.02, *I^2^* = 92%; Table 2, Fig. 2). However, in the allelic model, comparison of Lys with Glu allele generated a non-significant 3% increased cancer risk (OR = 1.03, 95%CI: 0.94–1.13, *P* = 0.52, *I^2^* = 86%; Table 2). Among the 53 case-control studies, 51 studies of Asians, 1 study of Africans [17], 1 study of mixed population [17]. When we restricted analyses to Asians, no change in OR occurred for either models (data not shown).

**Table 2:** Stratification analyses of the ALDH2 Glu504Lys polymorphism on cancer risk.

**Variables**	**N^a^**	**Sample size**	**Lys vs. Glu**			**Lys+ vs. Glu/Glu**		
		**case/control**	**OR (95% CI)**	***P***	***I*^2^ (%)**	**OR (95% CI)**	***P***	***I*^2^ (%)**
Total	53	16774/32060	1.03 (0.94–1.13)	0.52	86	**1.20 (1.03–1.39)**	**0.02**	92
Cancer type								
UADT	32	11187/19909	1.08 (0.95–1.23)	0.22	90	**1.39 (1.11–1.73)**	**0.004**	94
Colorectal	9	2082/4043	0.92 (0.79–1.08)	0.31	62	0.90 (0.75–1.08)	0.26	56
Hepatocelluar	5	729/879	0.93 (0.70–1.23)	0.61	61	0.99 (0.74–1.32)	0.95	51
Breast	3	1324/1756	0.96 (0.84–1.10) ^b^	0.53^b^	0	0.97 (0.82–1.14) ^b^	0.70^b^	0
Lung	2	1105/1803	1.12 (0.98–1.28)	0.11	NA	1.03 (0.77–1.37)	0.85	63
Pancreatic	2	347/3670	1.04 (0.87–1.23) ^b^	0.69^b^	0	1.14 (0.92–1.42) ^b^	0.24^b^	0
Country								
China	18	6193/8581	0.97 (0.84–1.12)	0.66	83	1.02 (0.83–1.26)	0.85	87
Japan	27	8217/21046	1.08 (0.95–1.22)	0.23	87	**1.38 (1.12–1.71)**	**0.003**	93
Others	8	2364/2433	0.99 (0.87–1.13) ^b^	0.93^b^	33	0.99 (0.86–1.13) ^b^	0.85^b^	0
Study design
PB	21	6635/11339	1.01 (0.89–1.16)	0.83	79	1.08 (0.90–1.29)	0.39	83
HB	30	8881/17767	1.02 (0.91–1.14)	0.72	84	**1.23 (1.02–1.49)**	**0.03**	91
Combined	2	1258/2954	1.34 (0.82–2.17)	0.24	87	1.87 (0.70–5.01)	0.21	95
Sample Size^c^
<300	36	5374/13859	1.01 (0.91–1.13)	0.82	72	1.18 (0.99–1.39)	0.06	81
>300	17	11400/18201	1.06 (0.91–1.24)	0.45	93	1.23 (0.94–1.62)	0.13	97

^a^: Number of studies.

^b^: Fix-effects model was used when *I* ^2^ <50%; otherwise, random-effects model was used.

^c^: Stratified according to subjects >300 in both case and control groups or not.

Combined, studies conducted on both population- and hospital-based control group; NA, not available.

### Subgroup analysis

**Figure 3:**
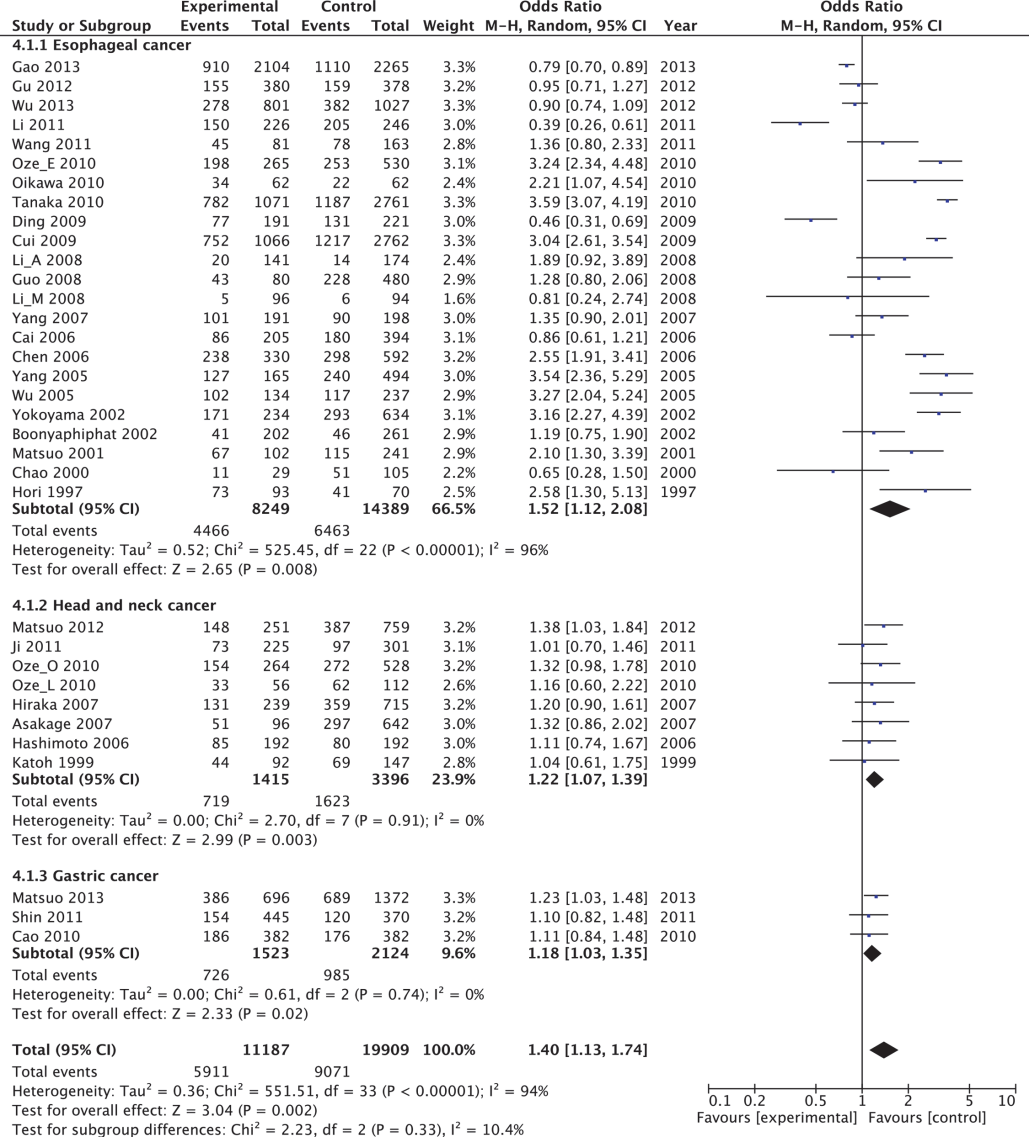
Forest plot of UADT cancer risk associated with the ALDH2 Glu504Lys polymorphism in position-specific analyses. Individuals with the variant allele (504Lys) had 52% increased risk of esophageal cancer (OR = 1.52, 95%CI: 1.12–2.08, *P* = 0.008), 22% risk of head and neck cancer (OR = 1.22, 95%CI: 1.07–1.39, *P* = 0.003) and 18% risk of gastric cancer (OR = 1.18, 95%CI: 1.03–1.35, *P* = 0.02).

In this meta-analysis, five cancer types were addressed: 32 studies focused on UADT cancer [17,19–21,23,24,27–51], 9 studies on colorectal cancer [16,18,52–58], 5 studies on hepatocellular carcinoma [22,26,59–61], 3 studies on breast cancer [62–64], 2 studies on lung cancer [25,65], and 2 studies on pancreatic cancer [16,66]. A significantly increased risk of UADT cancer (OR = 1.39, 95%CI: 1.11–1.73, *P* = 0.004, *I^2^* = 94%; Table 2) was observed in individuals with the Lys+ genotypes. Furthermore, according to the position of the tumor located, we performed position-specific analyses in the UADT cancer subgroup. The results indicated that individuals with the variant allele (504Lys) significantly increased 52% risk of esophageal cancer (OR = 1.52, 95%CI: 1.12–2.08, *P* = 0.008, *I^2^* = 96%; Fig. 3), 22% risk of head and neck cancer (OR = 1.22, 95%CI: 1.07–1.39, *P* = 0.003, *I^2^* = 0%; Fig. 3) and 18% risk of gastric cancer (OR = 1.18, 95%CI: 1.03–1.35, *P* = 0.02, *I^2^* = 0%; Fig. 3). However, the magnitude of association in genotypic models was weakened for digestive track cancers: colorectal cancer (OR = 0.90, 95%CI: 0.75–1.08, *P* = 0.26, *I^2^* = 56%; Table 2), hepatocellular cancer (OR = 0.99, 95%CI: 0.74–1.32, *P* = 0.95, *I^2^* = 51%; Table 2), pancreatic cancer (OR = 1.14, 95%CI: 0.92–1.42, *P* = 0.24, *I^2^* = 0%; Table 2) and breast cancer (OR = 0.97, 95%CI: 0.82–1.14, *P* = 0.70, *I^2^* = 0%; Table 2), lung cancer (OR = 1.03, 95%CI: 0.77–1.37, *P* = 0.85, *I^2^* = 63%; Table 2).

Of the 53 case-control studies, 27 studies were performed in Japan [16,20,24,26,27,30,36,37,40,41,44,45,47,48,50,51,54–59,61,63,65,66], 18 studies were performed in China [18,19,21,22,28,29,31,32,35,38,39,42,43,46,49,52,53,60], 8 studies were performed in other countries [17,23,25,33,34,62,64]. We found individuals from Japan with the Lys+ genotypes had a significant 38% increased cancer risk (OR = 1.38, 95%CI: 1.12–1.71, *P* = 0.003, *I^2^* = 93%; Table 2). However, we did not observe any significant associations among China (Lys vs. Glu: OR = 0.97, 95%CI: 0.84–1.12, *P* = 0.66, *I^2^* = 83%; Lys+ vs. Glu/Glu: OR = 1.02, 95%CI: 0.83–1.26, *P* = 0.85, *I^2^* = 87%; Table 2) or other countries (Lys vs. Glu: OR = 0.99, 95%CI: 0.87–1.13, *P* = 0.93, *I^2^* = 33%; Lys+ vs. Glu/Glu: OR = 0.99, 95%CI: 0.86–1.13, *P* = 0.85, *I^2^* = 0%; Table 2).

In the view of study design, of which 21 were population-based [16–18,21,22,25,28,29,32,35,38,39,43,44,47,51,53,54,60], 30 were hospital-based [19,20,23,24,26,27,30,31,33,34,36,40,42,45,46,48–50,52,55–59,61–66] and 2 studies were conducted on both population-based and hospital-based control group [37,41]. The magnitude of association in population-based studies was significantly weakened for genotypic model (OR = 1.08, 95%CI: 0.90–1.29, *P* = 0.39, *I^2^* = 83%; Table 2). Meanwhile, the magnitude of association in hospital-based studies was not significantly changed (Lys vs. Glu: OR = 1.02, 95%CI: 0.91–1.14, *P* = 0.72, *I^2^* = 84%; Lys+ vs. Glu/Glu: OR = 1.23, 95%CI: 1.02–1.49, *P* = 0.03, *I^2^* = 91%; Table 2).

In order to control for the difference of sample size, we chose the size of 300 in both case and control groups as the cut-off, 17 studies were conducted with subjects >300 [20,25,27–29,31,33,35–37,42,52,54,62–65]. However, no significant association was found in either model (Table 2).

### Test of heterogeneity

In the pooled analysis, we have found heterogeneities in allelic model comparison (Lys *vs* Glu: *P*_heterogeneity_ <0.00001, *I* ^2^ = 86%) and genetic model comparison (Lys+ *vs* Glu/Glu: *P*_heterogeneity_<0.00001, *I* ^2^ = 92%). A random effects model was performed in these analyses. Then we performed subgroup analysis based on cancer type, country, study design and sample size and assessed the source of heterogeneity for genetic model comparison (Lys+ *vs* Glu/Glu). As a result, cancer type (*χ* ^2^ = 10.05, df = 4, *P* = 0.04) and country (*χ* ^2^ = 7.18, df = 2, *P* = 0.03) but not study design (*χ* ^2^ = 1.86, df = 2, *P* = 0.39) or sample size (*χ* ^2^ = 0.09, df = 1, *P* = 0.76) were the significant sources of heterogeneity.

### Sensitivity analysis and Publication bias

Influence analysis was conducted by repeating the meta-analysis while excluding the studies that were not in HWE or the *P*_HWE_ values were not available. The estimated pooled odds ratio did not change, suggesting that the results are stable. Furthermore, when we conducted cancer-specific and size-specific sensitivity analyses, we found the magnitude of association in genotypic models was significantly strengthened in subgroup of colorectal cancer (OR = 0.85, 95%CI: 0.74–0.94, *P* = 0.02, *I^2^* = 23%; data not shown) and subjects <300 (OR = 1.23, 95%CI: 1.03–1.47, *P* = 0.02, *I^2^* = 80%; data not shown). Moreover, the estimated pooled odds ratio in other subgroups did not change, which suggested that the results of stratified analyses were also stable.

Lastly, to assess publication bias, we calculated the fail-safe number (N_fs_) at a significance level of 0.05 for each comparison. The N_fs_0.05 values for the comparison of Lys versus Glu (N_fs_0.05 = 2767), Lys+ versus Glu/Glu (N_fs_0.05 = 4998) were greater than the number of studies included in the meta-analysis.

## Discussion

To our knowledge, this is the first meta-analysis to evaluate the association between the Glu504Lys polymorphism of ALDH2 gene and the overall cancer risk. Our study suggests that individuals with the variant allele (504Lys) appear to be associated with an increased risk of cancer. Due to the prevalent of ALDH2 polymorphism in approximately half of East Asians but absent in Europeans and Africans [70] and possibility of population admixture that may potentially elevate type I error rate of association studies and lead to inconsistent results [71], we further excluded mixed populations and restricted analyses to Asians. However, no substantial change was observed, which confirmed the positive result of initial overall analyses. Genome-wide association (GWA) studies had also been previously conducted on the association of ALDH2 gene with cancer risks. McKay et al [67] reported the increased UADT cancer risk with the minor allele of rs4767364 in Europeans, which is similar to the UADT cancer risk effect observed for heterozygote rs671 carriers in Asians. Their results implicated the variant at 12q24 in UADT cancer susceptibility. With an elaborative genome-wide gene-environment interaction analysis, Wu et al [68] found that the most significant interaction region was for variants at 12q24 harboring ALDH2 and a joint analysis showed that alcohol drinkers carrying both risk alleles of ALDH2 and ADH1B had the highest risk of ESCC. Furthermore, Ioannidis et al [69] provided an overview of GWA-identified genetic associations with solid tumors since 2007 and showed the association between esophageal cancer and genetic variant rs671 with a median odds ratio (OR) of 1.67 (interquartile range = 1.58–1.76). The results from these GWA studies and our meta-analysis collectively suggest the importance of ALDH2 polymorphism carrying the susceptibility of cancer risks.

In the subgroup analysis by cancer type, significantly increased risk of UADT cancer with ALDH2 polymorphism was observed but no significant association was found among studies of other cancers (i.e., colorectal cancer, hepatocellular cancer, breast cancer, lung cancer and pancreatic cancer). In the UADT cancer subgroup, we further performed position-specific analyses and the results showed that individuals with the variant allele (504Lys) significantly increased 52% risk of esophageal cancer, 22% risk of head and neck cancer and 18% risk of gastric cancer. Some previous meta-analysis had reported the similarly elevated risks [72–74]. Furthermore, when we excluded the studies that were not in HWE or the *P*_HWE_ values were not available, interestingly, we found the effect of variant allele (504Lys) on colorectal cancer was contrary to that on UADT cancer. Recently, Zhao et al [9] had reported a similar result and put forward a possible explanation that the unpleasant symptoms resulting from high blood acetaldehyde levels after alcohol consumption may prevent the individuals with the variant allele (504Lys) from consuming alcohol and may keep them from developing alcoholism thus they have much lower chance to expose to the carcinogen acetaldehyde. However, as we could not perform subgroup analysis according to drinkers and non-drinkers to clarify the alcohol-genotype interaction, it is not possible to know whether the role of Lys+ genotypes is protective or not.

In the general population, the variant 504Lys allele is prevalent in Northeast Asian individuals (approximately 45% of Japanese, 31% of Chinese, 29% of Koreans and 10% of Thais) [75]. After stratified by country, significantly increased overall cancer risk was found in Japanese. However, no significant association was found in Chinese and populations from other countries. It may be uncommon for the same polymorphism playing different roles in cancer susceptibility in the same ethnic population. Oze et al [76] had collected four studies and showed that the Glu504Lys polymorphism had strong effect modification with alcohol drinking and alcohol drinking would increase the risk of esophageal cancer in the Japanese population. Meanwhile, a similar meta-analysis conducted in Chinese Han population had reached a similar conclusion [77]. However, data from the present study indicated that no association of this polymorphism with the overall cancer risk in Chinese. In addition, we had searched for the studies from other parts of Asia, such as South Asia, West Asia, Middle Asia, etc, but no data was available so far.

Heterogeneity is a potential problem when interpreting the results of a meta-analysis, and identifying the sources of heterogeneity is one of the most important goals of meta-analysis. In the present study, significant between-study heterogeneity in the pooled analyses of all included studies was found in both allelic and genetic models. To find the sources of heterogeneity, we performed subgroup analyses stratified by cancer type, country, study design and sample size. Our results indicated that the sources of heterogeneity were from cancer type and country, suggesting that the results of cancer-specific and country-specific analysis were reliable. Furthermore, if the distribution of genotypes in the control groups were not in HWE, the results of the genetic association studies might be spurious. Hence, we performed sensitivity analysis by excluding the studies that were not in HWE or the *P*_HWE_ values were not available. Except the cancer-specific analysis of colorectal cancer group and size-specific analysis of subject <300 group, the results were persistent and robust, suggesting that this factor had little effect on the overall estimates.

Despite the clear strength of our study including large sample sizes, some limitations of this meta-analysis should be mentioned. First, since the negative findings are usually difficult to get published or only published in some non-English journals, the ones that reported in other languages may bias the present results. Second, the present study was based on unadjusted ORs, and the confounding factors such as age may still bring some bias. Third, as the lack of sufficient original data, we could not conduct subgroup analysis according to drinking status that may influence the cancer risk. Forth, besides ALDH, activity of alcohol dehydrogenase (ADH) that is responsible for oxidation of ethanol to acetaldehyde can also play an important in the accumulation of acetaldehyde [3]; therefore, further study is needed to assess the independent and combined effect of ADH and ALDH polymorphisms.

In conclusion, this meta-analysis indicated that the Glu504Lys polymorphism of ALDH2 gene is a candidate for susceptibility to overall cancers, especially in esophageal cancer and among Japanese population. Moreover, due to the limitations mentioned above, well-designed studies taking into consideration of gene-gene and gene-environment interactions should be performed to confirm such associations.

## Supporting Information

S1 Table Detailed information of studies included in ALDH2 Glu504Lys polymorphism and cancer risk. (XLSX)

S1 Checklist Meta-analysis on Genetic Association Studies Checklist (PLOS ONE). (DOCX)

S2 Checklist PRISMA 2009 Checklist. (DOC)
